# Biochemical characterization of metabolism‐based atrazine resistance in *Amaranthus tuberculatus* and identification of an expressed *GST* associated with resistance

**DOI:** 10.1111/pbi.12711

**Published:** 2017-03-29

**Authors:** Anton F. Evans, Sarah R. O'Brien, Rong Ma, Aaron G. Hager, Chance W. Riggins, Kris N. Lambert, Dean E. Riechers

**Affiliations:** ^1^ Department of Crop Sciences University of Illinois at Urbana‐Champaign Urbana IL USA

**Keywords:** *Amaranthus tuberculatus*, herbicide metabolism, weed resistance, detoxification, oxidative stress, proteome, glutathione *S*‐transferase

## Abstract

Rapid detoxification of atrazine in naturally tolerant crops such as maize (*Zea mays*) and grain sorghum (*Sorghum bicolor*) results from glutathione *S*‐transferase (GST) activity. In previous research, two atrazine‐resistant waterhemp (*Amaranthus tuberculatus*) populations from Illinois, U.S.A. (designated ACR and MCR), displayed rapid formation of atrazine‐glutathione (GSH) conjugates, implicating elevated rates of metabolism as the resistance mechanism. Our main objective was to utilize protein purification combined with qualitative proteomics to investigate the hypothesis that enhanced atrazine detoxification, catalysed by distinct GSTs, confers resistance in ACR and MCR. Additionally, candidate *AtuGST* expression was analysed in an F_2_ population segregating for atrazine resistance. ACR and MCR showed higher specific activities towards atrazine in partially purified ammonium sulphate and GSH affinity‐purified fractions compared to an atrazine‐sensitive population (WCS). One‐dimensional electrophoresis of these fractions displayed an approximate 26‐kDa band, typical of GST subunits. Several phi‐ and tau‐class GSTs were identified by LC‐MS/MS from each population, based on peptide similarity with GSTs from *Arabidopsis*. Elevated constitutive expression of one phi‐class GST, named *AtuGSTF2*, correlated strongly with atrazine resistance in ACR and MCR and segregating F_2_ population. These results indicate that *AtuGSTF2* may be linked to a metabolic mechanism that confers atrazine resistance in ACR and MCR.

## Introduction

There are over 75 species in the genus *Amaranthus* found worldwide, including both monoecious and dioecious species (Mosyakin and Robertson, 2003). A dioecious species called waterhemp (*Amaranthus tuberculatus* (Moq.) Sauer var. *rudis* (Sauer) Costea & Tardif or syn. *A. rudis* Sauer) (Costea *et al*., 2005; Pratt and Clark, 2001) has become a major problem in the United States due to several ecological, biological, and genetic factors (Steckel, 2007). For example, waterhemp is difficult to selectively manage in maize and soybean (*Glycine max*) production systems because it is a summer annual with a prolonged germination period (Costea *et al*., 2005; Hartzler *et al*., 1999). In addition, the obligate outcrossing nature and dioecious biology of waterhemp facilitates the spread of genes conferring herbicide resistance via pollen flow throughout natural populations (Costea *et al*., 2005; Tranel *et al*., 2011). Multiple genes or alleles conferring resistance can occur within single waterhemp populations or individual plants due to strong herbicide selection pressures, resulting in multiple‐resistant phenotypes (Heap, 2016). For example, resistance to herbicides that inhibit 4‐hydroxyphenyl‐pyruvate dioxygenase (HPPD), protoporphyrinogen oxidase, acetolactate synthase (ALS), EPSP synthase, photosystem II (PS II) and the auxin herbicide 2,4‐D has been reported in waterhemp populations (Hausman *et al*., 2011; Heap, 2016; Patzoldt *et al*., 2005).

Atrazine is a commonly used herbicide for weed management in maize (LeBaron *et al*., 2011). PS II‐inhibiting herbicides such as atrazine inhibit the light reactions of photosynthesis by competing with plastoquinone for the Q_b_ binding site of the D1 protein (Fuerst and Norman, 1991; Hess, 2000), thus blocking the flow of electrons to cytochrome b_6_f and subsequently triggering the rapid formation of triplet chlorophyll followed by singlet oxygen in the presence of light (Krieger‐Liszkay, 2005; Triantaphylidès and Havaux, 2009) in sensitive dicots. In contrast, natural tolerance in maize and grain sorghum is due to the high constitutive activity of glutathione *S*‐transferase (GSTs) that can use atrazine as a substrate, leading to rapid metabolic detoxification in these crops (Timmerman, 1989). The most common mechanism conferring atrazine resistance in dicot weeds is an insensitive target‐site protein. A point mutation in the *psbA* gene (which encodes the D1 protein) frequently identified in atrazine‐resistant weeds results in a SER to GLY mutation at amino acid 264, which confers an approximate 1000‐fold level of resistance compared with sensitive biotypes (Devine and Preston, 2000; Hirschberg and McIntosh, 1983). By contrast, evolved resistance to atrazine in velvetleaf (*Abutilon theophrasti*) has been linked to elevated GST activity (Anderson and Gronwald, 1991; Gray *et al*., 1996). Similarly, GST‐based detoxification mechanisms have also been documented in several resistant grass weeds (Cummins *et al*., 2013; Reade *et al*., 2004; Yu and Powles, 2014). Rapid metabolism of atrazine in multiple‐resistant waterhemp resulted in a several hundred‐fold resistance level compared to atrazine‐sensitive plants (Evans, 2016).

GSTs are found in both plant and animals and are a widely studied class of primarily cytosolic (Mashiyama *et al*., 2014), dimeric enzymes mainly due to their detoxification abilities (Dixon *et al*., 2010; McGonigle *et al*., 2000; Wagner *et al*., 2002). Plant GST subunits belong to several different classes, including theta, zeta, lambda, phi, tau and glutathione‐dependent dehydroascorbate reductases (DHARs), based on sequence similarity, essential catalytic residues and immunological cross‐reactivity (Edwards and Dixon, 2005; Frova, 2006; Mashiyama *et al*., 2014). The most common subclasses of plant GSTs are the phi and tau classes (Labrou *et al*., 2015), although the relative proportions differ depending on species (Chi *et al*., 2011). Phi‐class GSTs were among the first GSTs shown to catalyse herbicide detoxification reactions in maize (Fuerst *et al*., 1993; Holt *et al*., 1995; Irzyk and Fuerst, 1993; Jepson *et al*., 1994).

Previous research demonstrated that atrazine resistance in two populations of waterhemp from Illinois (designated ACR and MCR; Hausman *et al*., 2011) results from non‐target‐site resistance (NTSR) mechanism(s), as indicated by the lack of a mutation in the *psbA* gene and rapid accumulation of a polar metabolite with the same retention time (via reverse‐phase HPLC) as a synthetic GSH‐atrazine standard in resistant populations (Ma *et al*., 2013). Therefore, we hypothesize that rapid formation of this metabolite results from increased GST activity in ACR and MCR compared to an atrazine‐sensitive population (WCS; Hausman *et al*., 2011) and that this increased activity results from either higher constitutive expression of GST(s) or the presence of novel GST isoforms with greater affinity towards atrazine. As a result, the objectives of this study were to (i) determine whether differences in GST activity exist between atrazine‐resistant and atrazine‐sensitive waterhemp populations, (ii) utilize ammonium sulphate (AMS) fractionation combined with GSH affinity chromatography to partially purify GSTs from each population and obtain peptide sequences, (iii) search a waterhemp transcriptome database to identify partial cDNA sequences encoding *GSTs* and (iv) determine whether expression of candidate *GST(s)* correlates with whole‐plant phenotypic responses to atrazine in the glasshouse, using an F_2_ population segregating for atrazine resistance (Huffman *et al*., 2015). Our results demonstrate that basal expression levels of a single candidate gene, named *AtuGSTF2*, correlate strongly with the atrazine‐resistant phenotype in ACR and MCR and segregating F_2_ population.

## Results

### GST activity towards atrazine in atrazine‐sensitive and atrazine‐resistant waterhemp populations

**Figure 1:**
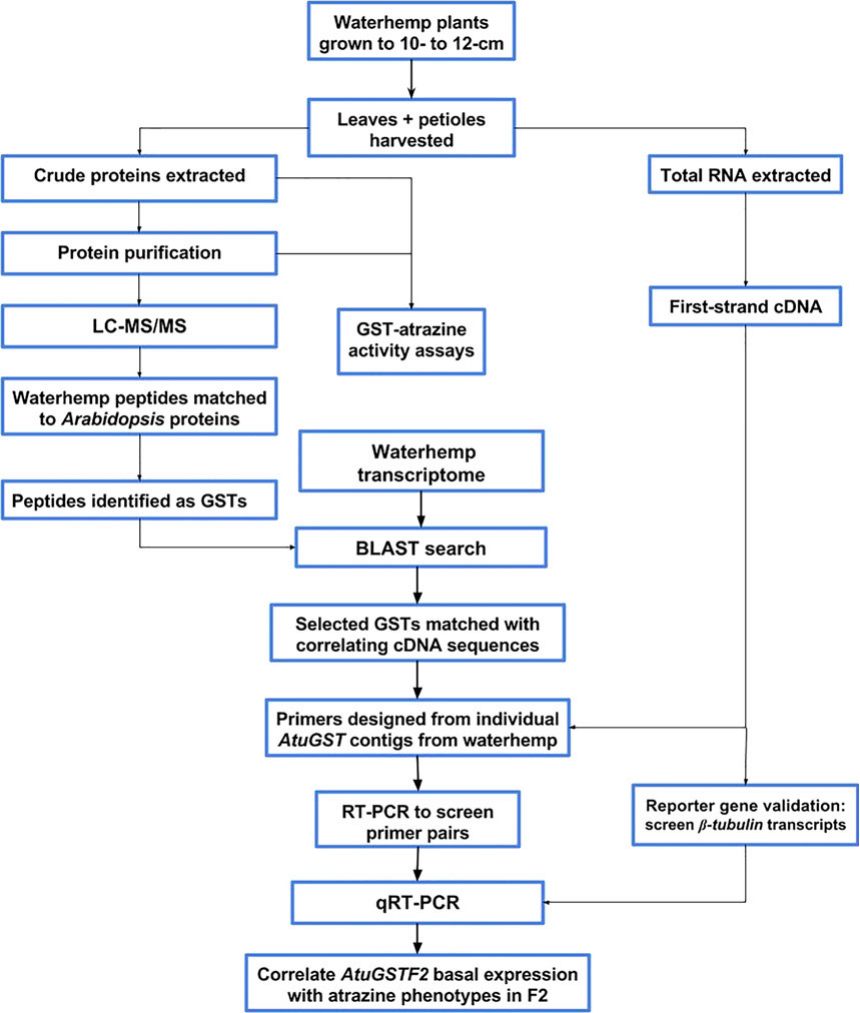
Flow diagram of GST purification procedures, protein identification by LC‐MS/MS,* AtuGSTF2* identification from a waterhemp transcriptome and gene expression analysis.

GST activity using atrazine as a substrate was measured in each waterhemp population (MCR, ACR, and WCS). The McLean County, Illinois (MCR), and Adams County, Illinois (ACR), populations are both resistant to atrazine due to rapid GST‐catalysed metabolism (Ma *et al*., 2013), while the Wayne County, Illinois (WCS), population is atrazine‐sensitive. Specific activities were compared among crude plant extracts (prepared from leaves and petioles), AMS‐precipitated fractions and GSH affinity‐purified fractions (Table 1). Differences in total or specific activity were not detected between crude protein extracts prepared from the resistant populations (ACR and MCR) compared to WCS. However, GST‐specific activities towards atrazine among the AMS fractions showed higher activity in ACR and MCR when compared to WCS, but a significant difference was not observed between ACR and MCR (Table 1). In order to further enrich and purify these fractions and attempt to isolate and identify unique or over‐expressed GST isozymes in MCR and ACR, a GST affinity purification method was incorporated following AMS purification (Figure 1). This purification scheme was based on previous methods used to investigate the role of specific GST isozymes in herbicide tolerance in etiolated maize, grain sorghum and wheat shoots (Gronwald and Plaisance, 1998; Irzyk and Fuerst, 1993; Riechers *et al*., 1997).

**Table 1:** GST activities partially purified from three different waterhemp (*Amaranthus tuberculatus*) populations, measured using atrazine as a substrate

Purification step	Total activity (pmol)			Specific activity (pmol/min/mg)			Activity yield			Fold purification		
	WCS	ACR	MCR	WCS	ACR	MCR	WCS	ACR	MCR	WCS	ACR	MCR
Crude	220.5	199.1	249.8	32.7 ± 1.4	36.0 ± 10.6	27.9 ± 0.9	100.0%	100.0%	100.0%	1.0	1.0	1.0
AMS	87.0	353.8	263.9	33.5 ± 5.5	86.9 ± 7.3	101.9 ± 8.6	39.5%	177.8%	105.6%	1.0	2.4	3.7
GSH	63.8	91.3	142.8	39.4 ± 9.1	163.1 ± 5.2	250.6 ± 8.0	28.9%	45.9%	57.2%	1.2	4.5	9.0

Crude, crude extract; AMS, 40%–80% ammonium sulphate cut; GSH, glutathione‐affinity column eluate.

**Figure 2:**
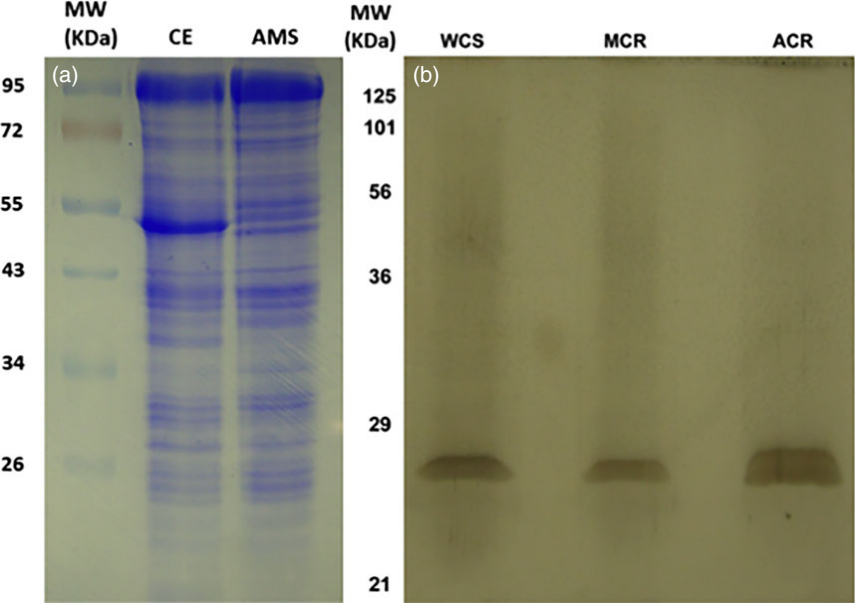
SDS‐PAGE of protein fractions throughout GST purification (Table 1; Figure 1), using the Laemmli (1970) buffer system and 12% resolving mini‐gels. (a) Coomassie blue‐stained crude protein extract (CE) and desalted proteins precipitated between 40% and 80% ammonium sulphate (AMS) fractions from the MCR population. Approximately 10 μg protein was loaded per lane. (b) Silver‐stained proteins of GSH affinity‐purified eluents from MCR, WCS and ACR. Approximately 40–80 ng protein was loaded per lane.

Specific activities increased significantly following each purification step. The largest fold‐increase in specific activity occurred during affinity purification with MCR, resulting in a *c.a*. 9‐fold purification relative to the crude extract (Table 1). Significant differences in specific activity between ACR or MCR and WCS were only determined in partially purified fractions (AMS and GSH affinity; Table 1). In addition, the specific activity yields measured in AMS fractions from ACR and MCR were greater than 100% (Table 1), consistent with the presence of GST inhibitors present in crude extract preparations. For example, this may occur when crude extracts contain many non‐GST proteins, pigments, metabolites or other cell debris that inhibit the GSH‐atrazine conjugation reaction *in vitro,* which are removed by AMS fractionation. After AMS precipitation, the 40%–80% fraction contained concentrated proteins that were readily soluble in buffer, with a significant removal of plant pigments, lipids and cell debris clearly visible. Partial protein purification was particularly evident when comparing the crude extract (CE) and AMS samples in Coomassie‐stained gels, where the dominant band in the CE fraction (presumably the large subunit of Rubisco; Figure 2a) at approximately 53 kDa is absent in the AMS fraction. GST‐specific activities measured in these fractions displayed a trend that was expected based on prior atrazine metabolism assays in these populations (Ma *et al*., 2013), with each resistant population having higher specific activity than WCS (Table 1).

GSH affinity‐purified fractions from each population showed a single, broad band at approximately 26 kDa when analysed by SDS‐PAGE and silver staining (Figure 2b), which is within the typical range (23–32 kDa) of plant GST subunits (Edwards and Dixon, 2005; Li *et al*., 2013) with few extraneous bands. However, these affinity‐purified fractions were presumed to include a heterogeneous mixture of GSTs and other cytosolic proteins (Figure 2b) with the ability to bind GSH (*e.g*. GSH reductase; Gill *et al*., 2013). Specific activity of these fractions followed the same pattern as with the AMS fractions (Table 1). However, fold‐purification levels for ACR and MCR (4.5 and 9.0, respectively, relative to crude extracts) were lower than previously reported in cereal crops (Gronwald and Plaisance, 1998; Irzyk and Fuerst, 1993; Riechers *et al*., 1997; Timmerman, 1989) for affinity‐purified GSTs.

### Qualitative LC‐MS analysis and identification of peptides from waterhemp GSTs

**Figure 3:**
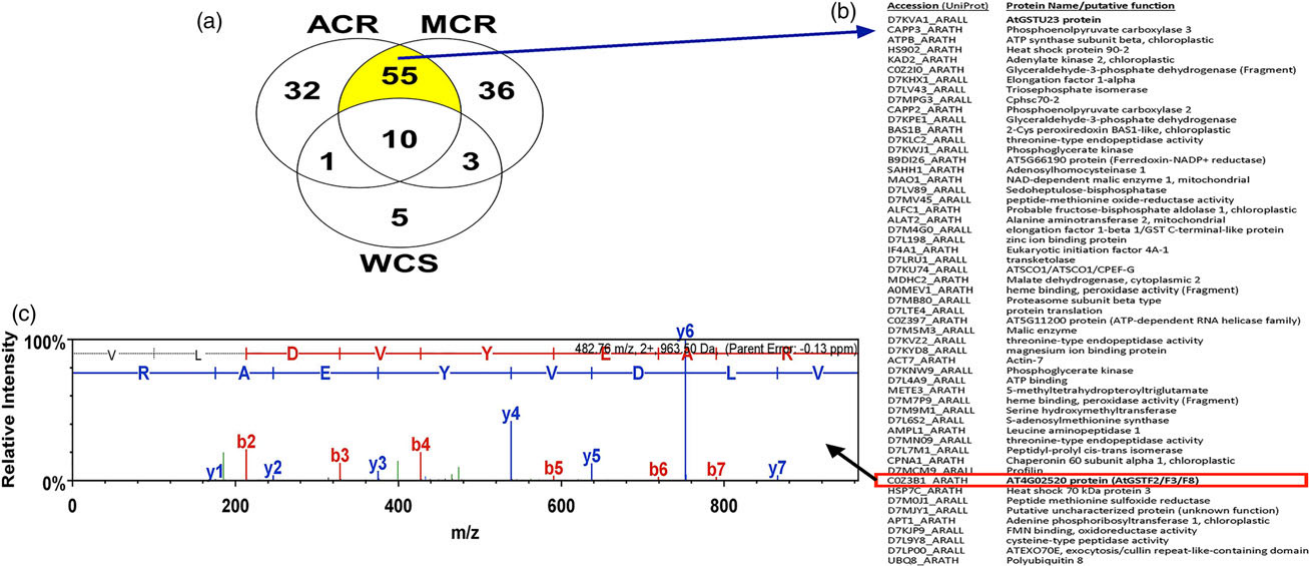
(a) Venn diagram displaying proteins unambiguously detected in representative GSH affinity fractions (Figure 2) from ACR, MCR and WCS populations, determined by searching *Arabidopsis thaliana* and *A. lyrata* protein databases (UniProtKB). In total, 55 proteins were shared between ACR and MCR. (b) Tentative identification of the 55 proteins from ACR and MCR, including the *Arabidopsis* accession identifiers and protein name/putative function. (c) Fragment ion spectra of the peptide primary sequence VLDVYEAR from ACR and MCR extracts with complete y‐ion series assignments, identified with sub‐ppm mass accuracy, assigned to protein AT4G02520 (*i.e*. AtGSTF2/F3/F8).

Excised SDS‐PAGE gel slices containing the approximate 26‐kDa protein bands from each population were subjected to protease digestion, LC‐MS analysis and subsequent peptide identification via alignment with the *Arabidopsis* reference genome. From the 142 *Arabidopsis* sequence matches obtained from one representative experiment (Figure 3a), proteins were initially screened for reported molecular weights within the typical range of plant GST subunit masses (*i.e*. 23–32 kDa) and approximate size of the excised protein bands (Figure 2b). Based on protein identification probability scores and unique peptide sequences, several phi‐class and tau‐class GSTs were subsequently identified from each waterhemp population. The Venn diagram lists the distribution of matched proteins among the MCR, ACR and WCS samples, with proteins only identified in MCR and ACR highlighted and described further (Figure 3a).

The waterhemp peptide sequence KVLDVYEARL identified from MCR and ACR digests (Figure 3a) matched an *Arabidopsis* protein annotated in GenBank as AtGSTF2 (Gene ID: AT4G02520), a phi‐class GST involved in various hormone and stress responses (Lieberherr *et al*., 2003; Smith *et al*., 2003, 2004) and binding of defence compounds (Dixon *et al*., 2011), and was the most probable match among all GSTs (Figure 3b). However, this diagnostic peptide is also found in AtGSTF3 and AtGSTF8 proteins (Table S1). The waterhemp peptide sequence RFWADYIDKK provided a unique and second strongest protein match among GSTs (Figure 3b); it was annotated in GenBank as AtGSTU23 (accession D7KVA1_ARALL), a tau‐class GST. Two additional tau‐class GSTs were identified from peptides that were present only in ACR. The peptide SPILLEMNPVHKK uniquely matched AtGSTU25, and the peptides SSLLLEMNPVHK and RIWAVKGEEQEAAK uniquely matched AtGSTU24.

Using these *Arabidopsis* GST protein sequences (identified using ACR and MCR peptides; Figure 1), best‐matching cDNA sequences were then identified from a waterhemp transcriptome database (Riggins *et al*., 2010), which resulted in five contigs encoding partial phi‐ or tau‐class waterhemp *GSTs* (Table S1). Five primer pairs (Table S2) were designed for RT‐PCR to amplify the largest possible section of each contig (from 200–800 bp), which were named based on the putative GST subclass (described in Edwards *et al*., 2000). *Atu* (*A. tuberculatus*) was chosen to identify the species name for these five waterhemp *GSTs* to prevent confusion with *Arabidopsis thaliana*. Each of the five primer pairs generated amplicons of the predicted size from each waterhemp population except for *AtuGSTF3*, which was not investigated further.

### RT‐qPCR analysis of *AtuGST* expression in MCR, ACR and WCS populations

RT‐qPCR was performed to determine whether the four remaining *AtuGST* genes differed in constitutive expression. Each of the four *AtuGST* cDNAs was amplified, and three of the four showed a single peak during melting‐curve analysis, indicating that a single amplicon was formed. *AtuGSTF1* was subsequently excluded from further RT‐qPCR analysis due to the nonspecific formation of multiple PCR amplicons. Transcript levels of *AtuGSTF2*,* AtuGSTU1* and *AtuGSTU2* were then determined in ACR or MCR relative to their corresponding expression in WCS using a stably expressed β‐tubulin reference gene (Table S1).

**Figure 4:**
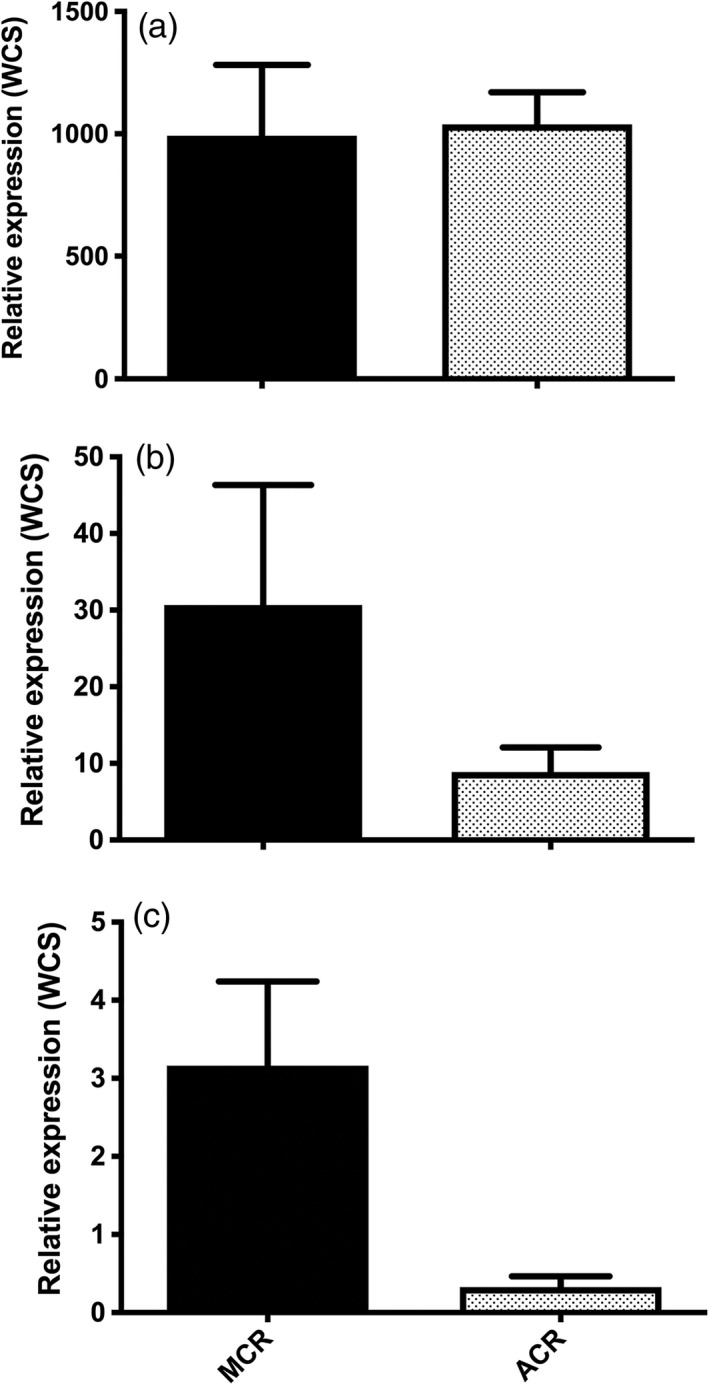
RT‐qPCR assays conducted for each target *AtuGST* gene using gene‐specific primers (Table S3). Dissociation curves for each reaction were analysed to ensure only one replicon was amplified. Constitutive expression of (a) *AtuGSTF2*, (b) *AtuGSTU1* and (c) *AtuGSTU2* in MCR and ACR quantified relative to expression of the corresponding *AtuGST* in WCS using the 2‐^∆∆^ ^CT^ method (Livak and Schmittgen, 2001) and β‐tubulin (Tables S1, S3) as a constitutively expressed control. Bars represent the standard error of the mean.

*AtuGSTF2* transcript levels in ACR and MCR were approximately 1000‐fold higher than in WCS, but a significant difference between transcript levels in ACR and MCR was not detected (Figure 4a). Significant differences were not detected in *AtuGSTU1* transcript levels among populations (Figure 4b). In contrast, transcript levels of *AtuGSTU2* were significantly higher in MCR than in ACR, but not between MCR and WCS or ACR and WCS (Figure 4c). Although not statistically different, a trend was observed where the abundance of *AtuGSTU1* and *AtuGSTU2* transcripts in MCR was higher than in WCS (Figure 4b–c). However, it is important to note that absolute expression levels of these two tau‐class GSTs were much lower (about 30–300 fold lower for *AtuGSTU1* and *AtuGSTU2*, respectively) when compared with *AtuGSTF2* expression levels in ACR and MCR (Figure 4a).

Higher constitutive expression of *AtuGSTF2* in ACR and MCR relative to WCS suggests that this gene (or allele) may contribute to higher levels of GST activity (Table 1) and rapid metabolism of atrazine (Ma *et al*., 2013) in these populations. As transcript abundance of *AtuGSTF2* in ACR and MCR (relative to WCS) was the only case where a clear pattern or association existed between basal expression and phenotype (Figure 4), *AtuGSTF2* was utilized as a candidate gene to further investigate its constitutive expression patterns in an F_2_ population of waterhemp (MCR x WCS) segregating for atrazine resistance as an incompletely dominant, qualitative trait (Huffman *et al*., 2015).

### Phenotyping atrazine responses, constitutive *AtuGSTF2* expression and genotyping in a segregating F_2_ population

**Figure 5:**
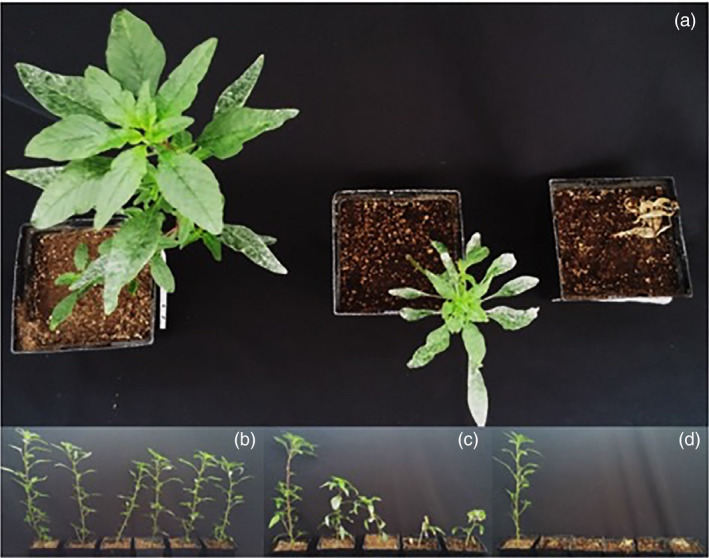
Whole‐plant responses of representative F_2_ lines, 12 days after treatment (DAT) with foliar‐applied atrazine. Waterhemp seedlings (10–12 cm tall) were treated with atrazine at the discriminatory rate of 14.4 kg/ha, including crop oil concentrate (1% v/v) and liquid ammonium sulphate (2.5% v/v) as adjuvants. (a) Top‐down view of treated plants, from left to right: homozygous (RR) resistant, heterozygous (Rr) resistant and sensitive (rr) phenotypic responses (12 DAT). (b) Side view of typical RR response at 12 DAT. (c) Side view of typical Rr response at 12 DAT. (d) Side view of typical rr response at 12 DAT (i.e. complete death). For the bottom panels (b‐d), the plant on the far left is an untreated control.

Due to the large degree of variability at the highest atrazine rate tested (28.8 kg/ha), a discriminatory rate (14.4 kg/ha) was determined as optimal for distinguishing between resistant genotypes. In order to determine whether constitutive expression of *AtuGSTF2* also correlated with phenotypic responses in the F_2_ population (Huffman *et al*., 2015), 10‐ to 12‐cm plants were treated with foliar‐applied atrazine at this rate. Treated plants revealed significant phenotypic differences among segregating F_2_ lines, as shown in Figure 5a and further described below. Plants from several F_2_ lines that rapidly developed healthy, new green tissue following atrazine treatment and were as tall as nontreated controls were tentatively assigned a homozygous (RR) atrazine‐resistant genotype (Figure 5b). By comparison, plants from numerous F_2_ lines that developed less green meristematic tissue than RR lines following atrazine treatment, were stunted, and did not grow significantly taller after application were tentatively assigned a heterozygous (Rr) atrazine‐resistant genotype (Figure 5c). Plants from several F_2_ lines died at this discriminatory rate within 7 days after treatment and were assigned an atrazine‐sensitive (rr) genotype (Figure 5d).

Dry weight reductions and constitutive *AtuGSTF2* expression (relative to WCS) in 10 representative lines from the F_2_ population are summarized in Table 2. Phenotypic responses resulting from atrazine treatment at 14.4 kg/ha correlated strongly with basal expression of *AtuGSTF2* (Table 2). In general, dry weight (biomass accumulation) and expression data followed the same trend, where dry weights were much higher in putative RR plants (lines 11 and 22), mainly because these plants developed a significant amount of green tissue following atrazine treatment (Figure 5b). Furthermore, lines 10, 21, 23, 31 and 32 displayed intermediate dry weights and *AtuGSTF2* expression values (Table 2), consistent with a putative Rr genotype. By comparison, rr lines did not accumulate biomass following atrazine treatment and eventually died at this rate (Figure 5d; Table 2).

**Table 2:** Phenotypes of selected segregating F_2_ lines, grouped according to dry weight (as a per cent of the untreated control) or *AtuGSTF2* expression (relative to β‐tubulin; *AtuBTUB1*)

F_2_ Line	Dry weight (% of untreated control)	Statistical grouping	*AtuGSTF2* relative expression	Statistical grouping	Atrazine genotype
22	54.1 (±7)	A	5742 (±2219)	A	RR
11	59.6 (±14)	A	5515 (±2244)	A	RR
21	33.9 (±5)	B	2546 (±1313)	BC	Rr
32	29.1 (±7)	BC	4233 (±1831)	AB	Rr
10	25.7 (±2)	CD	1008 (±489)	CD	Rr
31	20.0 (±4)	D	2998 (±1951)	BC	Rr
23	13.7 (±3)	E	1089 (±516)	CD	Rr
26	10.9 (±3)	E	4 (±1)	D	rr
27	5.1 (±1)	E	9 (±5)	D	rr
7	2.7 (±1)	E	2 (±1)	D	rr

These whole‐plant results are consistent with the corresponding *AtuGSTF2* expression levels in each line, which were extremely high in RR and Rr lines by comparison with rr lines (Table 2), ranging from approximately 200‐fold (line 10) to 1140‐fold (line 22) higher. When considering all RR and Rr lines together, the mean *AtuGSTF2* expression value of 3304 units is 661‐fold greater than the mean expression of 5 units for all rr lines. The large difference in *AtuGSTF2* expression between resistant and sensitive genotypes indicates the robustness of utilizing this constitutively expressed gene as a marker for identifying metabolic‐based atrazine‐resistant genotypes in this F_2_ population, as well as in MCR, ACR and possibly other NTSR waterhemp populations yet to be analysed.

Exceptions to the overall strong association were noted, as evidenced by a weak fit within a discrete statistical category when considering both dry weight reductions and their *AtuGSTF2* expression (*e.g*. lines 23 and 32; Table 2). Based on the statistical groupings and categorization of phenotypic responses displayed in the glasshouse (Table 2), however, genotypes assigned for the atrazine‐resistance trait in these lines are consistent with the F_2_ population segregating for resistance in a 3 : 1 ratio (Huffman *et al*., 2015); *viz*. more Rr lines were identified than either homozygous genotype (RR or rr) from the original 32 F_2_ lines investigated.

### Sequence analysis of individual *AtuGSTF2* amplicons from parent waterhemp populations and individual F_2_ lines

Sequencing results from a total of 11 individual RT‐PCR products (185‐bp) indicated the presence of identical transcripts (*AtuGSTF2.2*) in each Rr or RR F_2_ line tested (5 total; lines 10, 11, 22, 31 and 32). In addition, four individual amplicons derived from the MCR population (four different plants) possessed this same sequence, which differed from the original waterhemp transcriptome sequence (*AtuGSTF2.1*) by one conservative amino acid change within this region (Figure S1a). In contrast, analysis of two different amplicons from two rr genotypes (lines 7 and 26) and four individual amplicons from the WCS population (two different plants) revealed three sequences; the *AtuGSTF2.2* allele (from all resistant plants tested) and the *AtuGSTF2.1* allele, plus an additional allele (*AtuGSTF2.3*) found only in line 26 (Figure S1a).

Sequence variants of *AtuGSTF2* identified from the limited amount of amplicons are consistent with the dioecious, outcrossing nature of waterhemp. Sequence analysis of additional F_2_ lines and individual clones may yield more allelic variants of the genes listed in Figure S1a–b. However, the lack of polymorphisms among all *AtuGSTF2* amplicons sequenced from atrazine‐resistant plants thus far (15 total) suggests a single haplotype containing the R allele (*AtuGSTF2.2*) in MCR and the F_2_ population. This haplotype might occur if higher constitutive expression in resistant genotypes, as compared to sensitive genotypes, results from genetic variability that exists within the promoter (or untranslated regions) several kb upstream of the *AtuGSTF2* gene (Mahmood *et al*., 2016). Further analysis is required, however, because the 185‐bp *AtuGSTF2* sequence represents *c.a*. 30% of the coding region (Figure S1c).

Sequence alignment of AtGSTF2 with the partial amino acid sequence of AtuGSTF2.1 (Figure S1c) revealed 67% identity, although this is a preliminary comparison as the AtuGSTF2.1 sequence only represents a portion of the full‐length protein. The sequence of maize ZmGSTF2, a phi‐class GST (previously called maize GST II, GST IV, or GST‐27), was included for comparison because its involvement in herbicide detoxification and stress responses has been well documented (Edwards *et al*., 2000; Holt *et al*., 1995; Irzyk and Fuerst, 1993; Jepson *et al*., 1994). Comparison of the full‐length sequences of ZmGSTF2 and AtGSTF2 showed 39% identity and comparison of ZmGSTF2 with the partial AtuGSTF2.1 sequence revealed 44% identity, which is within the expected range for interspecific comparisons of GSTs within a subclass (Labrou *et al*., 2015; Yang *et al*., 2009). It is important to note, however, that the *c.a*. 30% of AtuGSTF2.1 aligns closely with the N‐terminus of AtGSTF2 (Figure S1c). Plant GST sequences from the same subclass are strongly conserved in the N‐terminal domain of the protein, which typically corresponds with Exon 1 of the genomic sequence (Frova, 2006; Labrou *et al*., 2015), relative to the C‐terminus. Interestingly, the diagnostic phi‐class waterhemp GST peptide KVLDVYEARL is present in AtGSTF2 and ZmGSTF2, although the ZmGSTF2 sequence contains one conservative amino acid change (Figure S1c).

## Discussion

NTSR mechanisms to herbicides in weeds (such as enhanced herbicide detoxification) have drawn great interest in recent years, particularly in grass weed species (Cummins *et al*., 2013; Gaines et al., 2014; Reade *et al*., 2004; Yu and Powles, 2014). However, metabolic resistance in dicots is not well characterized and remains markedly under‐explored, particularly regarding the underlying biochemical mechanisms, enzymes and specific genes in these species (Anderson and Gronwald, 1991; Gray *et al*., 1996; Ma *et al*., 2013). Atrazine resistance in dicots is typically conferred by a point mutation in the plastidic target‐site gene *psbA* (encoding the D1 protein in PS II), leading to decreased atrazine binding (reviewed by Devine and Preston, 2000). In contrast to previous research aimed at sequencing *psbA*, our primary goal was to characterize total and specific GST activities from atrazine‐resistant MCR and ACR populations and compare with activities in the atrazine‐sensitive population WCS, thereby following up on previous atrazine metabolism findings (Ma *et al*., 2013).

In spite of a significant enrichment in specific activity in MCR and ACR (and WCS to a lesser extent) protein extracts throughout the purification scheme, fold‐purification levels were much lower than previously reported in cereal crops (Gronwald and Plaisance, 1998; Irzyk and Fuerst, 1993; Riechers *et al*., 1997; Timmerman, 1989) for affinity‐purified GSTs. These lower fold‐purification levels in our research may have resulted from use of photosynthetic tissues instead of etiolated seedling, shoot or coleoptile tissues from cereals (Irzyk and Fuerst, 1993; Riechers *et al*., 1997) or loss of activity during sample processing following initial extract preparation through GSH affinity purification as protein fractions become more dilute. However, these results establish a framework for continued mechanistic investigations of evolved resistance to atrazine, other pesticides or metabolism of environmental/endogenous toxins by GSTs in weedy *Amaranthus*. In addition, these findings pave the way for new biotechnology applications aimed towards overcoming metabolic resistance in weedy plants, as described in detail below.

### Possible underlying mechanisms for elevated basal *AtuGSTF2* expression in waterhemp

*AtuGSTF2* displayed higher constitutive expression in both atrazine‐resistant waterhemp populations as well as in resistant F_2_ lines segregating as a single‐gene trait. Greater transcript abundance of the *AtuGSTF2* gene may contribute to elevated GST activity (Table 1) and higher levels of GSH‐atrazine metabolites formed in ACR and MCR compared with WCS (Ma *et al*., 2013). Thus far, higher GST‐specific activities with atrazine quantified in partially purified ACR and MCR protein extracts can only be associated with higher constitutive expression of *AtuGSTF2*. Further experiments are required, however, to obtain the entire open reading frame for expression and biochemical analyses of the recombinant AtuGSTF2 enzyme (with atrazine as substrate) because plant genomes contain dozens of *GST* genes and isozymes (Chi *et al*., 2011; McGonigle *et al*., 2000; Riechers *et al*., 2010) that contribute to total activity. From the standpoint of gene regulation, the potential for induction of *AtuGSTF2* expression by atrazine pretreatment in waterhemp should be examined in future research.

Higher basal expression levels of *AtuGSTF2* could be due to a mutation, insertion or varying degrees of methylation in the *AtuGSTF2* promoter or untranslated regions (Mahmood *et al*., 2016), an alteration in a DNA‐binding protein, or a protein regulating mRNA stability. *GSTs* are unevenly dispersed throughout plant genomes (Dong *et al*., 2016; Lan *et al*., 2009; ) or found in clusters of duplicated genes (Soranzo *et al*., 2004; Xu *et al*., 2002), thus promoting gene evolution and functional diversification (Kaltenegger and Ober, 2015; Liu *et al*., 2013). Alternatively, a mutation in a single transcription factor (TF) protein that binds to *GST* promoters could coordinately activate the expression of multiple *AtuGST*s. An analogous situation occurs with the maize Opaque‐2 TF protein and DNA‐binding One Zinc Finger (Dof) protein OBP1 (Noguero *et al*., 2013), which together regulate the transcription of numerous zein genes (Li *et al*., 2015; Vicente‐Carbajosa *et al*., 1997). Interestingly, an OBP1 protein identified in *Arabidopsis* regulates expression of the *GST6* gene (renamed *AtGSTF8*; Wagner *et al*., 2002) in a similar manner (Chen *et al*., 1996). Additional genomic sequence analyses of *AtuGSTF2* from resistant and sensitive plants are required to fully understand the resistance mechanism, however, because the alignments in Figure S1 only represent an estimated 30% of the *AtuGSTF2.1* coding sequence.

### Transcript profiling techniques may reveal the entire waterhemp GSTome

In weedy grass species without entire genomic sequences available, RNAseq has recently been utilized for the characterization of evolved resistance mechanisms (Chen *et al*., 2017; Duhoux *et al*., 2015; Gaines *et al*., 2014). Global transcriptional analyses of distinct genotypes identified from the segregating F_2_ population (Table 2), using the available waterhemp genome and transcriptome (Lee *et al*., 2009; Riggins *et al*., 2010) along with the sequenced grain amaranth (*Amaranthus hypochondriacus*) genome and transcriptome (Sunil *et al*., 2014) as references, could more accurately determine the number of expressed *GST* genes (Chi *et al*., 2011; Labrou *et al*., 2015) in waterhemp and may enable further confirmation of the specific *AtuGST* gene(s) correlating with resistance. If RNAseq analyses indicated that *AtuGSTF2* (or other metabolic enzymes) are the major genetic factor(s) correlating with resistance then further biochemical and molecular characterizations could be performed with the full‐length AtuGSTF2 coding region, such as transgenic plant analysis with an atrazine‐sensitive dicot such as *Arabidopsis* or tobacco. In addition, genomic cloning of *AtuGSTs* and subsequent bioinformatic comparisons with other herbicide‐detoxifying plant *GSTs* (Cummins *et al*., 2011; Edwards *et al*., 2000; Frova, 2006) may reveal regulatory motifs or sequence variations between resistant and sensitive waterhemp plants (Mahmood *et al*., 2016).

### New biotechnology applications towards improving resistant‐weed management

Basal expression levels of *AtuGSTF2* could be used as a molecular marker for screening putative resistant waterhemp populations to exclude or confirm metabolic resistance to atrazine. A similar approach was utilized following the discovery of a key mutation associated with metabolic resistance to dichlorodiphenyl‐trichloroethane (DDT) in the mosquito *Anopheles funestus* (Riveron *et al*., 2014). A genomewide transcriptional analysis was conducted in which the most highly upregulated gene was identified as a *GST* (termed *GSTe2*), which was confirmed to confer resistance to DDT and cross‐resistance to pyrethroid insecticides through transgenic expression in sensitive *Drosophila*. The molecular basis for resistance resulted from both quantitative and qualitative mechanisms; increased expression in resistant mosquitos combined with a point mutation in the wild‐type *GSTe2* gene in which LEU was substituted for PHE (Riveron *et al*., 2014).

In addition to improved resistance screening methods, the coding sequence of *AtuGSTF2* could be utilized to engineer targeted gene knockout strategies such as RNAi directed to a specific *Amaranthus GST* (Yu and Powles, 2014) or to synthesize new chemical inhibitors of herbicide‐detoxifying GSTs (Cummins *et al*., 2013; Lamoureux and Rusness, 1986; Ma *et al*., 2016). RNAi‐based knockdown techniques have been used successfully in insect systems where insecticide‐detoxifying P450s have been targeted (Bautista *et al*., 2009; Zhu *et al*., 2016), thus regaining activity of the insecticide in resistant populations. Polynucleotide‐based gene knockdown systems are also being generated to overcome herbicide resistance in weeds (Sammons *et al*., 2015), which to date have been targeted primarily towards herbicide target‐site proteins. However, additional knowledge of specific herbicide‐detoxifying isozymes in weeds, such as those belonging to large, multigene GST and P450 families, provides a new opportunity to regain herbicide activity in multiple‐resistant weeds. The findings presented herein support the conclusion that increased basal expression of a specific herbicide‐detoxifying GST is associated with atrazine resistance in MCR and ACR, which may ultimately confer atrazine resistance, but might also lead to innovative and integrated weed management strategies.

## Experimental procedures

### Plant populations

Waterhemp populations used in these experiments were from McLean County (atrazine‐resistant [MCR]), Adams County (atrazine‐resistant [ACR]) or Wayne County (atrazine‐sensitive [WCS]) Illinois, USA, as described previously (Hausman *et al*., 2011; Ma *et al*., 2015). Seeds were germinated in 12 × 12‐cm containers with a commercial potting medium in a growth chamber using previously described methods (Ma *et al*., 2013). Waterhemp seedlings (2‐cm) were transferred to 80‐cm^3^ containers in the glasshouse containing the same medium. When seedlings reached 4‐cm, plants were transferred to 950‐cm^3^ pots with a 3 : 1 : 1 : 1 mixture of potting mix:soil:peat:sand, and a slow‐release fertilizer was then added. Plants were then harvested (10‐ to 12‐cm in height) for subsequent protein and enzyme extraction. Growth chamber and glasshouse conditions were maintained at 28 °C/22 °C day/night with 16/8‐h photoperiod. Growth chamber light was provided by incandescent and fluorescent bulbs delivering 550 μmol/m^2^/s photon flux at plant canopy level. Natural glasshouse light was supplemented with mercury halide lamps, with a minimum output of 500 μmol/m^2^/s photon flux at plant canopy level.

### Tissue homogenization, crude protein extraction and enrichment of GST activity

Plants from each population were harvested as described above and stems were removed due to interference with mechanical disruption. All leaves (including the attached petioles) were frozen in liquid nitrogen and pulverized. Polyvinylpolypyrrolidone (7.5% w/v) was added along with 3 mL of protein extraction buffer (100 mm Tris‐HCl (pH 7.0), 1 mm DTT, 1 mm Na_2_EDTA) per gram fresh weight. Samples were then filtered through three layers of cheesecloth and centrifuged at 3000 ***g*** for 30 min at 5 °C to obtain a clarified crude extract.

Clarified extracts were used to prepare 40%–80% AMS fractions based on protein solubility in water at 5 °C. Initial experiments determined more than 90% of GST activity (with atrazine as substrate) precipitated in this range, which is similar to previously published reports of plant GST purification (Edwards and Dixon, 2005; Riechers *et al*., 1997; Smith *et al*., 2004). After slowly adding AMS during fractionation, samples were stirred for 20 min at 5 °C then centrifuged at 3000 ***g*** for 30 min at 5 °C. Pelleted material from the 40% cut was discarded as well as the soluble fraction following the 80% cut. The remaining pellet from the 80% cut was then desalted in protein storage buffer (100 mm Tris‐HCl (pH 7.0), 1 mm DTT, 1 mm Na_2_EDTA) using 7K MWCO Zeba™ Spin Desalting Columns (Thermo‐Fisher Scientific, Waltham, MA, USA) and stored at −80 °C.

### GSH affinity purification and SDS‐PAGE analysis

Desalted and concentrated protein samples from each population obtained after AMS precipitation were further purified by immobilized GSH‐Sepharose affinity chromatography (GSTrap™ FF columns, General Electric‐Life Sciences, Pittsburgh, PA, USA) using a Biologic DuoFlow fast‐protein liquid chromatography (FPLC) system (Bio‐Rad Laboratories, Hercules, CA, USA). A modification of the manufacturer's recommended protocol for affinity chromatography was developed and utilized. The column was equilibrated with 5 volumes of binding buffer (140 mm NaCl, 10 mm NaHPO_4_, 1.8 mm KHPO_4_, and 2.7 mm KCl, pH 7.4). Protein samples (0.1–3.0 mg) were then loaded on the column and washed with 10 column volumes of binding buffer at a flow rate of 5 mL/min. Bound proteins were eluted with 10 volumes of elution buffer (50 mm Tris‐HCl (pH 8.0), 10 mm GSH, 1 mm DTT) at a flow rate of 5 mL/min and samples were then immediately desalted into protein storage buffer as described above. Protein concentration was measured with a Nanodrop spectrophotometer using bovine serum albumin (BSA) as standard. SDS‐PAGE was conducted according to the method of Laemmli (1970) utilizing 12% resolving mini‐gels (Bio‐Rad, Hercules, CA, USA) to examine purity of each fraction. Proteins were visualized with either Coomassie Brilliant blue or silver stained according to the manufacturer's protocol (FASTSilver™ kit, G‐BioSciences, St. Louis, MO, USA).

### GST‐atrazine activity assay

A modified version of a previously published protocol was used to measure GST activity towards atrazine (Gronwald and Plaisance, 1998). Reactions (300 μL) contained 50 μm ^14^C‐atrazine (2 mCi/mmol specific activity), 2 mm GSH, 100 mm Na‐citrate (pH 6.5), 1 mm 2‐mercaptoethanol, 0.2 mg/mL BSA and protein with a final concentration ranging from 0.05–1 mg/mL. Negative controls to determine nonenzymatic conjugation of GSH with atrazine were assayed for each replicate by replacing the protein fraction with extraction buffer. After incubation for 5 min at 30°C, reactions were initiated by addition of ^14^C‐atrazine and incubated at 30°C for 30 min. Initial experiments under these conditions determined that product formation was linear until this time point. Reactions were terminated with 50 μL acetic acid and then partitioned against 900 μL methylene chloride to separate parent atrazine from the atrazine‐GSH conjugate. Radioactivity in a 200‐μL aliquot of the aqueous phase was quantified by liquid scintillation spectrometry (LSS). Enzymatic conjugation rates were determined by averaging radioactivity found in the aqueous phase of the negative control reactions (*i.e*. no protein added) and subtracting this amount from the total activity measured in the experimental reactions. Specific activity was determined based on protein concentration and nonenzymatically corrected radioactivity quantified in the aqueous phase by LSS. Units of GST activity are reported as pmol of GSH‐conjugated atrazine per minute per mg protein. GST activity results represent the combined data from two independent experiments with three technical replications per assay.

### Sample preparation, LC‐MS/MS and bioinformatic analysis of peptide sequences

Coomassie‐stained bands, representing proteins from each population eluting from GSH affinity columns (~100 ng), were manually excised from one‐dimensional SDS‐PAGE gels. Protein fractions were analysed using nanoLC‐MS/MS by the Stanford University Mass Spectrometry Laboratory (https://mass-spec.stanford.edu/proteomics) as described below. Gel slices were diced into 1‐mm^2^ sections, rinsed multiple times with 50 mm ammonium bicarbonate and reduced with 5 mm DTT in 50 mm ammonium bicarbonate at 55 °C for 30 min. Residual solvent was removed and alkylation performed using 10 mm propionamide in 50 mm ammonium bicarbonate for 30 min at 23 °C. Gel pieces were rinsed with 50% acetonitrile and 50 mM ammonium bicarbonate and dried under vacuum for 5 min. Protein digestion was performed with trypsin/LysC (Promega Corporation, Madison, WI, USA) overnight at 37 °C. Tubes were centrifuged, and the supernatant including peptides was collected. Further peptide extraction was performed by the addition of 60% acetonitrile, 39.9% water, 0.1% formic acid and incubation for 10–15 min. Peptide pools were dried, concentrated and reconstituted for further analysis.

Digested peptides were injected onto a C_18_ reversed‐phase analytical column (20 cm in length; 100 μm internal diameter). HPLC was performed with an Eksigent nanoLC at a flow rate of 600 nL/min using a linear gradient from 4% (mobile phase B) to 35% (mobile phase B). Mobile phase A consisted of 0.1% formic acid/water and mobile phase B was 0.1% formic acid/acetonitrile. All data were collected using an LTQ Orbitrap Velos mass spectrometer set to acquire data in a data‐dependent fashion, selecting and fragmenting by CID the most intense precursor ions optimized to maximize duty cycle. An exclusion window of 60 s was used to improve proteomic depth and multiply charged cations required for triggering MS/MS. Data were analysed using Preview and Byonic v2.6.49 (ProteinMetrics) and ported into Scaffold_4.7.2 (Proteome Software, Inc.; http://www.proteomesoftware.com/products/scaffold), as well as custom in‐house tools for data analysis developed in MATLAB. Using Scaffold, reference *Arabidopsis* proteomes (NCBI and UniProtKB) were searched with peptide sequences obtained from each waterhemp population using a reverse‐decoy strategy, and all data filtered and presented at a 2% false discovery rate where two peptides per protein were required. Proteins that contained similar peptides but could not be differentiated based on MS/MS analysis alone were grouped to satisfy the principles of parsimony.

*Arabidopsis* proteins containing matching peptide sequences were sorted based on protein identification probability. Protein matches (based on amino acid identity) were then screened first by excluding proteins outside the target mass range for typical plant GST subunits (23–32 kDa) and subsequently omitting proteins without an annotated function in the database. Accession numbers for identified AtGSTs were obtained through the NCBI Basic Local Alignment Search Tool (BLAST). Protein sequences from these accessions were then used to search waterhemp genome (Lee *et al*., 2009; https://www.ncbi.nlm.nih.gov/sra/SRX005233) and transcriptome (Riggins *et al*., 2010; https://www.ncbi.nlm.nih.gov/sra/SRX018843) databases to identify best‐matching DNA sequences. Gene‐specific primers (Table S2) were designed from each matching waterhemp contig (Table S1) for subsequent expression analyses and sequence confirmation (depicted in Figure 1).

### Total RNA extraction, RT‐PCR and initial contig analysis

Total RNA was extracted from waterhemp tissues and prepared using previously described methods (Riechers *et al*., 2003). Total RNA concentrations were determined using a NanoDrop spectrometer and rRNA quality was confirmed by visual analysis in agarose‐formaldehyde gels. First‐strand cDNA synthesis was performed using the Maxima H‐Minus cDNA synthesis kit (Thermo‐Fisher Scientific, Waltham, MA, USA) following the manufacturer's protocol using 500 ng total RNA. The following parameters were then used for RT‐PCR, with 1 μL of first‐strand cDNA reaction: initial denaturing at 95 °C for 4.5 min, then 30 amplification cycles consisting of 95 °C for 50 s, 56 °C for 55 s and 72 °C for 1 min, followed by final extension at 72 °C for 8.5 min. RT‐PCR products were visualized with 1% agarose gels stained with ethidium bromide.

Semi‐quantitative RT‐PCR (Riechers *et al*., 2003) was utilized first to determine whether the five matched *AtuGST* contigs identified in the waterhemp transcriptome (Riggins *et al*., 2010) were expressed in each waterhemp population. Five primer pairs (Table S2) were designed to amplify the largest possible section of each *GST* contig (ranging from 200–800 bp). A β*‐tubulin* reference gene (*AtuBTUB1*; Table S1) with highest similarity to an *Arabidopsis* tubulin beta‐7 chain (*AtTUB7*; Gene ID: AT2G29550) was selected from among several annotated tubulin contigs from waterhemp (Lee *et al*., 2009; Riggins *et al*., 2010) based on initial RT‐PCR screening. Additionally, crosses between primers matching the same contig (*i.e*. forward primer for *AtuGSTF1* with reverse primer for *AtuGSTF2*; Table S2) were used to confirm that individual waterhemp contigs originated from the same mRNA template.

### RT‐qPCR analysis of *AtuGST* expression in MCR, ACR and WCS populations

RT‐qPCR was performed with total RNA isolated from each waterhemp population using the same tissues and growth stage as described previously. Gene‐specific primers were redesigned to specifically amplify the *AtuGSTs* identified (based on the SYBR Green protocol for RT‐qPCR; Table S3), although original primers for specifically amplifying *AtuGSTF2* were retained. Stable, constitutive expression of *AtuBTUB1* was demonstrated under the experimental conditions and waterhemp growth stage used in these studies as determined by <1‐fold magnitude of differences in CT values. Primer efficiencies for RT‐qPCR ranged from 95% to 99% for *AtuGSTF2* and *AtuBTUB1* amplifications from cDNA (Table S3). RT‐qPCR was conducted using the 7900 HT Sequence Detection System (PerkinElmer, Applied Biosystems, Waltham, MA, USA) and reactions performed in 20 μL volumes following the manufacturer's protocol (Syber^®^ Green RNA‐to C_T_™ 1‐Step Kit; Applied Biosystems, Waltham, MA, USA). The protocol was as follows: 48 °C for 30 min, 95 °C for 10 min, 40 cycles at 95 °C for 15 s, 60 °C for 1 min and a melting curve at 95 °C for 15 s, 60 °C for 15 s and 95 °C for 15 s. Dissociation curves for each reaction were analysed to ensure only one replicon was amplified. Gene expression in each sample was calculated relative to transcript levels in WCS and the *AtuBTUB1* reference gene using the 2^−∆∆C^_T_ method (Livak and Schmittgen, 2001).

For analysis of *AtuGSTU1*,* AtuGSTU2* and *AtuGSTF2* expression in the MCR, ACR and WCS populations, expression data represent the combined results from three independent experiments (*i.e*. biological replicates) with three technical replications for each RNA sample. ANOVA followed by Tukey's multiple comparison test (*P* = 0.05) was conducted to determine significant differences in gene expression. For analysis of *AtuGSTF2* expression in segregating F_2_ lines, experiments were independently conducted twice with three technical replications per RNA sample. Data from each experiment were combined and *AtuGSTF2* relative expression values were analysed by LSD (*P* = 0.1) using PROC GLM in SAS (Release 9.2) to determine significant differences among F_2_ lines.

### Phenotyping of whole‐plant responses to atrazine in an F_2_ population

A total of 32 F_2_ plants were randomly selected from the original MCR × WCS cross (Huffman *et al*., 2015). Vegetative clones derived from these original F_2_ ‘parent’ plants were used to study segregation of whole‐plant responses and gene expression due to the large amount of genetic variability in waterhemp (Ma *et al*., 2013; Steckel, 2007). When sufficient clones had been generated to represent each F_2_ line (Ma *et al*., 2015), a dose–response study was conducted to compare the response of the 32 F_2_ lines to foliar‐applied atrazine. When plants reached 10–12 cm in height, they were treated with atrazine at rates evenly spaced along a 3.16 log scale (Hausman *et al*., 2011), ranging from 3.2 g/ha to 10 000 g/ha, and included 1% crop oil concentrate (COC) as a spray adjuvant. Control plants were treated with water plus COC only.

This initial study broadly determined which F_2_ lines were sensitive or resistant to atrazine. GR_50_ values for the sensitive lines ranged from 25 to 69 g/ha, significantly lower than the maximum field‐use rate of 2.2 kg/ha (Ma *et al*., 2016). However, complete death was never achieved in atrazine‐resistant lines, and estimated GR_20_ values for all resistant lines were greater than the field‐use rate. A discriminatory rate was then determined to distinguish between RR and Rr plants. Atrazine rates ranged from 1.2 kg/ha to 28.8 kg/ha and included 1% COC and 2.5% liquid AMS as spray adjuvants. Due to the large degree of variability at the highest rate tested, the discriminatory rate of 14.4 kg/ha was determined for distinguishing between resistant genotypes. By comparison, a much lower rate of atrazine (985 g/ha) had been used previously to distinguish between resistant (RR and Rr) and sensitive genotypes in this F_2_ population (Huffman *et al*., 2015), but a different growth medium and nutrient system was utilized compared with the methods described herein. Experiments were independently conducted at least twice with five replications per treatment. Aboveground biomass was harvested at 12 DAT, dried in an oven at 65 °C, and dry weight data were combined and analysed by LSD (*P* = 0.1) using PROC GLM in SAS (Release 9.2) to determine significant differences among F_2_ lines.

### Sequence analysis of individual *AtuGSTF2* and *AtuBTUB* amplicons from cDNA

Total RNA was extracted from nontreated waterhemp tissues (using methods described earlier) from different F_2_ lines, with at least three representative lines from each putative genotype. Gene‐specific primers (Table S3) were used to amplify *AtuGSTF2* and *AtuBTUB* alleles using methods described earlier for RT‐qPCR. RT‐PCR products were purified directly from each reaction using the QIAquick™ PCR Purification Kit (Qiagen Inc., Valencia, CA, USA). Purified amplicons were then ligated into a pCR™4‐TOPO cloning vector and transformed into competent *E. coli* cells (TOPO TA™ Cloning Kit, Invitrogen, Waltham, MA, USA). Plasmids were purified using the I‐Blue Mini Plasmid Kit (IBI Scientific, Peosta, IA, USA) and submitted for sequencing. Amplicons were sequenced from a total of seven different F_2_ lines (two RR, three Rr and two rr), plus the original MCR and WCS populations, originating from at least two different colonies per transformation reaction.

## Conflict of interest

The authors declare no conflict of interest.

## Supporting information


**Figure S1.** Partial cDNA sequences of *AtuGSTF2* and *AtuBTUB1* alleles expressed in several F_2_ lines from the segregating population described by Huffman *et al*. (2015).

**Table S1.** Proposed gene nomenclature and GenBank accession numbers (for sequenced allelic variants) for waterhemp cDNAs (*AtuGSTs* and *AtuBTUB1*), best‐matched *GSTs* from *Arabidopsis*, and waterhemp contig information. Contigs are derived from a waterhemp transcriptome database (Riggins *et al*., 2010; https://www.ncbi.nlm.nih.gov/sra/SRX018843).

**Table S2.** Primers used for waterhemp contig analysis and initial screening approach via semi‐quantitative RT‐PCR.

**Table S3.** Primers used for candidate gene expression analysis via RT‐qPCR.
