# Supplementary material for: Biochemical characterization of metabolism‐based atrazine resistance in Amaranthus tuberculatus and identification of an expressed GST associated with resistance
Source: Plant Biotechnol J. 2017 Mar 29;15(10):1238–49. doi: 10.1111/pbi.12711 (PMC5595711; doi:10.1111/pbi.12711)

**Figure S1** Partial cDNA sequences of *AtuGSTF2* and *AtuBTUB1* alleles expressed in several F_2_ lines from the segregating population described by Huffman *et al.,* (2015)

Deduced amino acid alignments of different (a) AtuGSTF2 or (b) β-tubulin (AtuBTUB1) proteins, encoded by partial cDNAs. (c) Partial AtuGSTF2.1 sequence aligned with the corresponding region of the best-matched *Arabidopsis* protein (AtGSTF2) and maize ZmGSTF2 (all phi-class GSTs). Amino acids highlighted in gray in AtGSTF2 and ZmGSTF2 represent the corresponding region of AtuGSTF2 where the diagnostic peptide KVLDVYEAR was identified by LC-MS (Figure 3).


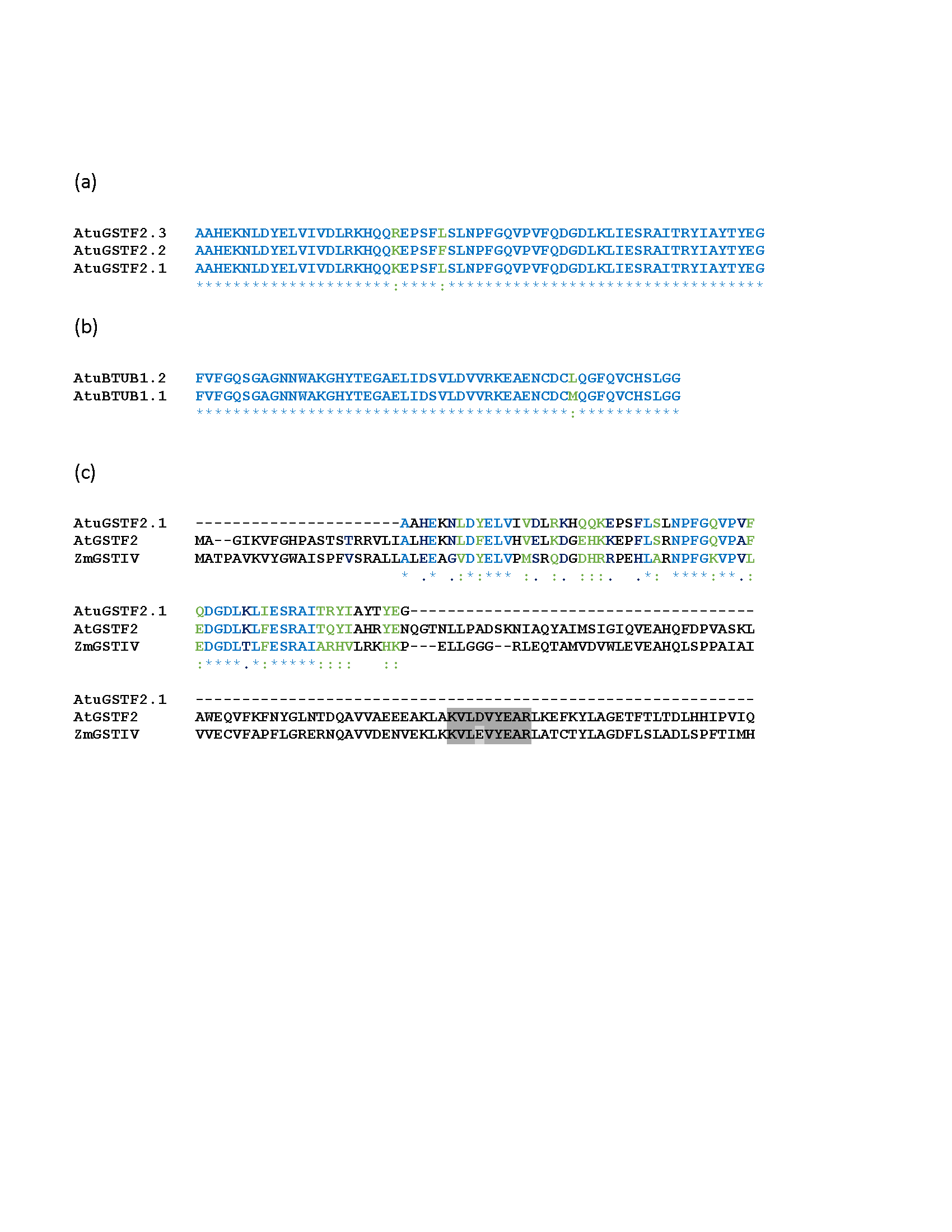

Supplement: Supplementary file 1 — Figure S1. Partial cDNA sequences of AtuGSTF2 and AtuBTUB1 alleles expressed in several F2 lines from the segregating population described by Huffman et al. (2015). [file PBI-15-1238-s003.doc]
