# Supplementary material for: Biochemical characterization of metabolism‐based atrazine resistance in Amaranthus tuberculatus and identification of an expressed GST associated with resistance
Source: Plant Biotechnol J. 2017 Mar 29;15(10):1238–49. doi: 10.1111/pbi.12711 (PMC5595711; doi:10.1111/pbi.12711)
Supplement: Supplementary file 2 — Table S1. Proposed gene nomenclature and GenBank accession numbers (for sequenced allelic variants) for waterhemp cDNAs (AtuGSTs and AtuBTUB1), best‐matched GSTs from Arabidopsis, and waterhemp contig information. Contigs are derived from a waterhemp transcriptome database (Riggins et al., 2010; https://www.ncbi.nlm.nih.gov/sra/SRX018843). [file PBI-15-1238-s002.doc]

**Table S1** Proposed gene nomenclature and GenBank accession numbers (for sequenced allelic variants) for waterhemp cDNAs (*AtuGSTs* and *AtuBTUB1*), best-matched *GSTs* from *Arabidopsis*, and waterhemp contig information

Contigs are derived from a waterhemp transcriptome database (Riggins *et al.* 2010; https://www.ncbi.nlm.nih.gov/sra/SRX018843).

| Gene designation | GenBank accession no. (waterhemp cDNAs) | Best matched *Arabidopsis* GST(s) | Matching waterhemp contig | Contig/accession size (bp) |
| --- | --- | --- | --- | --- |
| *AtuGSTF1* | N/A | AtGSTF2  AtGSTF3 | Contig 14479; cDNA | 305 |
| *AtuGSTF2* | KY196994 (*AtuGSTF2.1*)  KY196995 (*AtuGSTF2.2*)  KY196996 (*AtuGSTF2.3*) | AtGSTF2  AtGSTF3 | Contig 20117; cDNA | 185 |
| *AtuGSTF3* | N/A | AtGSTF2 | Contig 13931 rev; cDNA | 736 |
| *AtuGSTU1* | N/A | AtGSTU25  AtGSTU28  AtGSTU23 | Contig 12319; cDNA | 590 |
| *AtuGSTU2* | N/A | AtGSTU25  AtGSTU28  AtGSTU24 | Contig 14728; cDNA | 439 |
| *AtuBTUB1* | KY196997 (*AtuBTUB1.1*)  KY196998 (*AtuBTUB1.2*) | TUB7 | Contig 3713, cDNA | 157 |
