# Supplementary material for: Biochemical characterization of metabolism‐based atrazine resistance in Amaranthus tuberculatus and identification of an expressed GST associated with resistance
Source: Plant Biotechnol J. 2017 Mar 29;15(10):1238–49. doi: 10.1111/pbi.12711 (PMC5595711; doi:10.1111/pbi.12711)
Supplement: Supplementary file 3 — Table S2. Primers used for waterhemp contig analysis and initial screening approach via semi‐quantitative RT‐PCR. [file PBI-15-1238-s004.doc]

**Table S2** Primers used for waterhemp contig analysis and initial screening approach via semi-quantitative RT-PCR

| Gene | Forward Primer (5’-3’) | Reverse Primer (5’-3’) |
| --- | --- | --- |
| ***AtuGSTF1*** | GCTAATGAGAAAGAGCTTCAATAC | ACTTACTACTGGATCAAATTGG |
| ***AtuGSTF2***  ***AtuGSTF3*** | GCACCCAACGTGTATTAG  CGCCAAGCATTGACATGAGG | AGTAAGGGGTGTTCCTTG  AAACTCGCAATGGAGCTTGC |
| ***AtuGSTU1*** | TGAGAATAGTAGGAGAATTTGGAC | CTCAACCCATACTTCAATCTACC |
| ***AtuGSTU2*** | TCCTATCCCAAGATAAACTCAG | GGAGTATATTGATGAATATTGGAAAGG |
