# Supplementary material for: Biochemical characterization of metabolism‐based atrazine resistance in Amaranthus tuberculatus and identification of an expressed GST associated with resistance
Source: Plant Biotechnol J. 2017 Mar 29;15(10):1238–49. doi: 10.1111/pbi.12711 (PMC5595711; doi:10.1111/pbi.12711)
Supplement: Supplementary file 4 — Table S3. Primers used for candidate gene expression analysis via RT‐qPCR. [file PBI-15-1238-s001.doc]

**Table S3** Primers used for candidate gene expression analysis via qRT-PCR

| Gene | Forward Primer (5’-3’) | Reverse Primer (5’-3’) |
| --- | --- | --- |
| ***AtuGSTF1*** | GCTAATGAGAAAGAGCTTCAATAC | ACTTACTACTGGATCAAATTGG |
| ***AtuGSTF2*** | GCACCCAACGTGTATTAG | AGTAAGGGGTGTTCCTTG |
| ***AtuGSTU1*** | TCGAGTAGGGGAAACGCGGTA | CCAGACCCTGCCTCTAGCGA |
| ***AtuGSTU2*** | ACCCCAAGCAATGATTTTGGGACA | AGACACTAGAACAAGTGTTGGGAGA |
| ***AtuBTUB1*** | AGATTTTTCGCCCGGATAAC | TCCCATTCCAGATCCTGTTC |
